# Effects of Radix Linderae extracts on a mouse model of diabetic bladder dysfunction in later decompensated phase

**DOI:** 10.1186/s12906-019-2448-1

**Published:** 2019-02-04

**Authors:** Xufeng Yang, Dawei Lian, Pinglong Fan, Yifei Xu, Jing Wang, Fangjun Chen, Huanling Lai, Weiwen Jiang, Linjie Zhang, Ping Huang, Hongying Cao

**Affiliations:** School of Pharmaceutical Sciences, Guangzhou University of Chinese Medicine, Guangzhou, 51006 Guangdong China

**Keywords:** Diabetic bladder dysfunction, Later decompensated phase, Radix Linderae, High-fat diet, Streptozotocin

## Abstract

**Background:**

This study aimed to elucidate the effects and mechanisms of Radix Linderae (RL) extracts on a mouse model of diabetic bladder dysfunction (DBD), especially on later decompensated phase.

**Methods:**

Male C57BL/6J mice were intraperitoneally injected with streptozotocin (STZ) after 4 weeks of high-fat diet (HFD) feeding. DBD mouse models (later decompensated phase) were developed by 12-weeks persistent hyperglycemia and then treated with RL extracts for 4 weeks. During administration, the fasting blood glucose (FBG) test was performed once a week. Four weeks later, oral glucose tolerance test (OGTT), voided stain on paper (VSOP), and urodynamic alteration were explored. We also performed haematoxylin and eosin (H&E) and Masson’s trichrome staining to observe the histology of the bladder. Then, the contractile responses to α, β-methylene ATP, capsaicin (CAP), KCl and carbachol were measured. Moreover, qPCR assay was performed to analyse the bladder gene expression levels of M_3_ receptors and TRPV1.

**Results:**

The diabetic mice exhibited higher FBG, OGTT and urine production, and no substantial alteration was observed after RL treatment. Urodynamic test showed the maximum bladder capacity (MBC), residual volume (RV) and bladder compliance (BC), as well as the decrement of voided efficiency (VE) and micturition volume (MV), remarkably increased in the DBD mice. Furthermore, RL treatment significant improved urodynamic urination, with lower MBC, RV, and, BC, as well as higher VE and MV, as compared with the model groups. The wall thickness of the bladder and the ratio of smooth muscle/collagen remarkably increased, and RL could effectively attenuate the pathological change. The response of bladder strips to the stimulus was also reduced in the DBD mice, and RL treatment markedly increased the contraction. Furthermore, the gene expression levels of M_3_ receptors and TRPV1 were down-regulated in the bladders of the diabetic mice, whereas RL treatment retrieved those gene expression levels.

**Conclusions:**

RL extracts can improve the bladder voiding functions of the DBD model mice in later decompensated phase, and underlying mechanisms was associated with mediating the gene expression of M_3_ receptors and TRPV1 in the bladder instead of improving blood sugar levels.

**Electronic supplementary material:**

The online version of this article (10.1186/s12906-019-2448-1) contains supplementary material, which is available to authorized users.

## Background

Traditionally, diabetic bladder dysfunction (DBD), also known as diabetic cystopathy, was characterized by a series of lower urinary tract symptoms and the most common urologic complication of diabetes [1, 2]. DBD featured two temporal progression phases: an initial compensatory hypertrophic phase (occurred after the onset of diabetes and was characterised by bladder hypertrophy, remodelling, increased contractility and associated neurogenic changes) and a later decompensated phase (developed at later stages of diabetes and featured decreased peak voiding pressure) [2]. The International Diabetes Federation has estimated that 425 million people suffered from diabetes, whereas 212 million adults with diabetes are undiagnosed worldwide in 2017 (IDF Diabetes Atlas 8th Edition 2017) and the treatment of diabetes and its complications has brought a heavy burden to the society. Almost 50–80% of patients with diabetes were affected by DBD [3, 4], especially the later decompensated phase of DBD, which inflicts great pain to the patient.

Controlling blood glucose level was the foundation in diabetes treatment. However, controlling blood glucose failed to completely eliminate DBD. A clinical study reported that 48.0% diabetics had urinary incontinence and 22.5% patients had overactive bladder, even then patients who took oral hypoglycaemic drugs still experience such condition [5]. Corresponding therapeutic methods, such as drug administration, lower abdominal massage, catheterization, Kegel exercises, and surgical procedures, target different stages of DBD [6]. However, at present, the clinical requirement for effective treatment of DBD remained insufficient and more novel drugs were urgently demanded.

The function of urinary bladder contractility was regulated mainly by cholinergic and purinergic pathways [7, 8] and subordinately by other nerve pathways, such as adrenaline, tachykinins [9] and capsaicin (CAP) [10]. Other mediators of contraction are similarly important as acetylcholine, especially under pathological conditions.

In recent years, the use of traditional Chinese medicine and natural products in the treatment of diabetic and its complications has become noteworthy due to its effectiveness and less toxicity [11]. Radix Linderae (RL; named Wu-Yao in Chinese), the dried root of Lindera aggregata (Sims) Kosterm, was the main component of Suo Quan Wan, which has been shown to enhance bladder storage and contraction ability [12] and improve bladder voiding function based on the regulation of transient receptor potential vanilloid-1 (TRPV1) [13, 14]. Furthermore, RL has been used for centuries to combat frequent urination and other urinary bladder diseases [15–17]. Hence, we designed the experiment to explore the possible effects and the underlying mechanisms of RL on the treatment of DBD through the mediation of cholinergic and transient receptor potential vanilloid-1 (TRPV1) pathways [18]. Combination of high-fat diet (HFD) and streptozotocin (STZ) injection was used to establish a type 2 diabetes mellitus (T2DM) mouse model, which presented patients characteristic features of T2DM [19, 20].

## Methods

### Reagents and materials

Streptozotocin (STZ) was purchased from TOKU-E Co. Ltd. (Japan); HFD (45% fat) was purchased from Guangdong Medical Laboratory Animal Centre (China); Mecobalamin was purchased from Eisai Pharmaceutical Co. Ltd. (China); Roche dynamic blood glucose meter was purchased from HoffmannLa Roche Inc. (Switzerland); carbachol was obtained from Bausch & Lomb Incorporated, (China); α, β-methylene ATP was from Sigma Chemical Co. Ltd. (USA); CAP was purchased from Cayman Chemical Co. Ltd. (USA); Dispelling RT SuperMix and Talent qPCR PreMix (SYBR Green) were purchased from TIANGEN Technology Biology Co. Ltd. (China); All other reagents used were of analytical grade.

Radix Linderae (RL) was provided from Zhejiang Biological Engineering Co., Ltd. (China) and identified by Wenru Wu (the associate professor of School of Pharmaceutical Sciences, Guangzhou University of Chinese Medicine). Specification of this extract was provided in Table 1 and authenticated in accordance with the Chinese Pharmacopoeia (committee 2015) (Data was shown in Additional file 1) [21].

**Table 1:** Specification of Radix Linderae used in this study

Chinese names	Scientific name	Origin of region	Batch number	Extraction solvent
Wu-Yao	Radix Linderae	Zhejiang China	130,515	50% ethanol

### Animal model of type 2 diabetes and treatment

Seventy male C57BL/6J mice (6 weeks old, 18–22 g) were obtained from the Beijing Vital River Laboratory Animal Technology Co., Ltd., and housed in Experimental Animal Centre of Guangzhou University of Chinese Medicine at 25 ± 2 °C and exposed to a 12 h/12 h light–dark cycle, with free access to food and water. Then, the animals were randomly divided into two groups: group I (control mice, *n* = 10) and group II (type 2 diabetic mice, *n* = 60). Group I and II mice were fed with standard diet and HFD, respectively. After 4 weeks of HFD feeding, group II mice (fasted for 12 h, but water was allowed ad libitum) were injected intraperitoneally with STZ (100 mg/kg) for 4 times to induce type 2 diabetes (about 2 weeks), whereas group I mice were injected with the vehicle only (0.05 M citric acid, pH 4.3–4.5). After the last injection, the animals with fasting blood glucose (FBG) levels ≥189 mg/dl were selected and randomly divided into four groups: the model group (*n* = 10), positive group (Methycobal: 0.82 mg·kg^− 1^·d^− 1^, n = 10) high-dose RL (HRL) group (RL: 3.6 g·kg^− 1^·d^− 1^, n = 10) and low-dose RL (LRL) group (RL: 0.9 g·kg^− 1^·d^− 1^, n = 10). After 12 weeks feeding, all animal groups were oral gavage with distilled water, Methycobal, HRL and LRL for 4 weeks, respectively.

### The fasting blood glucose (FBG) and oral glucose tolerance test (OGTT)

During administration, FBG tests were performed once a week. After 4 weeks administration, all group mice (FBG and OGTT were measured at 9 o ‘clock after fasted for 12 h, but water was allowed ad libitum) were subjected to an OGTT. Glucose solution was administered by gavage to all groups at doses of 2 mg/g total body weight. One drop of blood was extracted from each tail of mice by glucometer (ACCU-CHEK Active) to determine glucose concentration. Briefly, measurements were performed out at 0, 15, 30, 60, and 120 min after glucose challenge (Formula: AUC_0-2h_ = (Bg0 + Bg15) × 0.5 ÷ 4 + (Bg15 + Bg30) × 0.5 ÷ 4 + (Bg30 + Bg60) × 0.5 ÷ 2 + (Bg60 + Bg120) × 0.5).

### Voided stain on paper (VSOP) analysis

After 4 weeks administration, the mice were placed individually in cages for 5 h (food and water were allowed ad libitum). Urine output was measured by evaluating the surface area of the stained VSOP paper. The collected papers were imaged under ultraviolet light to visualize urine output.

### Urodynamic test in vivo

Urodynamic studies were performed by using an urodynamic measuring device (Laborite Delphis 94-R01-BT, Canada) and microinjection pump. General anaesthesia was induced by intraperitoneally injecting of 25% urethane (2.0 mg/kg). The animals were in a dorsal recumbent position, and the bladder was surgically exposed via a midline abdominal incision. A 25-gauge needle was inserted into the dome of the bladder, which was connected via a 3-way stopcock to a pressure line at one end and a microinjection pump at the other. The pressure line was connected to a physiologic pressure transducer of the urodynamic measuring instrument. After the bladder was emptied, sterile room-temperature saline was infused at 3 ml per hour, and pumping was stopped when urine was observed at the external orifice of the urethra. The signals were recorded automatically by a computer. The parameters included the following: micturition volume (MV), maximum voiding pressure (MP), residual volume (RV), maximum bladder capacity (MBC), bladder compliance (BC), and voided efficiency (VE). RV was drained manually and measured by using a 1 ml syringe. MBC was calculated as infused volume immediately before detrusor contraction. BC was calculated as (MBC/MP) × 100%; VE was calculated as (MBC − RV) / MBC × 100%. Mean values of three voiding cycles of each mouse were used to eradicate any discrepancies. The assay was performed thrice for each mouse.

### Determination of plasma insulin

At the end of the urodynamic test experiment, the mice plasma was obtained by abdominal aortic blood collection. And then, plasma insulin levels were detected by radioimmunoassay (Iodine [1251] insulin radioimmunoassay kit, Beijing north biotechnology research institute Co. Ltd. (China)) according to the instructions.

### Histological test

The mice were euthanized by cervical dislocation, and the bladder was fixed in paraformaldehyde solution, sectioned by following the routine procedure, and embedded in paraffin. The processed bladder tissue was sectioned at 5 μm. The sections were stained with haematoxylin and eosin (H&E) and Masson’s trichrome and examined by a light microscopy. The bladder wall thickness was determined based on H&E images. The ratio of smooth muscle to collagen within the bladder smooth muscle layer wall was measured by using Masson’s trichrome-stained images. The above-mentioned parameters were assessed by image analysis software (Image-Pro Plus. 6.0).

### Assessment of bladder smooth muscle contractility in vitro

The mice were euthanized by cervical dislocation, and the urinary bladder was quickly removed at the level of the bladder neck. Then the bladder was placed in Kreb’s solution (composition: 140 mM Na^+^, 5.9 mM K^+^, 104.8 mM Cl^−^, 1.2 mM H_2_P0_4_^−^, 24.9 mM HCO_3_^−^, 2.6 mM Ca^2+^, 1.15 mM Mg^2+^, 1.15 mM SO_4_^2−^, and 10 mM glucose). Full-thickness longitudinal detrusor strips (0.7 to 1 mm × 5 mm) were prepared and transferred to tissue baths. The baths contained 5 ml of Kreb’s solution bubbled with a mixture of 95% O_2_ and 5% CO_2_ at 37 °C [22]. One end of each strip of detrusor was tied using silk sutures to a force transducer and the other to a fixed hook. Signals were monitored using the PowerLab system (AD Instruments Pty. Ltd., Australia). Each strip of tissue was set up under 0.5 g passive tension before the experiments were performed. Following balance for 60 min, contractile responses to stimulation of smooth muscles by α,β-methylene ATP (α,β-methylene ATP) (100 μM), CAP (10 μM) and potassium chloride (KCl: 120 mM) were measured, and a cumulative concentration–response curve to carbachol (Shandong Dr. Renfreida Pharmaceutical; China) was obtained by adding the agonist (10^− 8^ M to 10^− 5^ M) to the bath. After adding one stimulant to the bath, the strip was washed with Kreb’s solution thrice and allowed to balance for 45 min before adding another agonist. At the end of experiments, the weight of each strip was measured to normalize force data.

### Real-time PCR

Total ribonucleic acid (RNA) was extracted from the bladder by using Trizol reagent (Invitrogen, Rockville, MS USA) according to the instructions. The RNA samples were estimated by absorption at 260 and 280 nm. A260/A280 was used to check the purity, and the concentration of RNA was confirmed using the A260 values (Shimadzu BioSpec-nano, Japan). cDNA syntheses were obtained from the total RNA by using Fastking Gdna Dispelling RT SuperMix (TIANGEN, China), and a qPCR assay was performed using an ABI Prism 7500 system (Applied Biosystems, USA) with Talent qPCR PreMix (SYBR Green) (TIANGEN, China). Synthetic oligonucleotide primers were designed to amplify cDNA for the genes encoding the M_3_ receptors, TRPV1 and GAPDH. Primer pairs were listed in Table 2. The reaction program was as follows: 95 °C for 30 s followed by 39 cycles at 95 °C for 3 s and 60 °C for 34 s. Results are expressed as mRNA levels of each gene studied, and each result was normalized according to GAPDH expression [23].

**Table 2:** Primers sequence of GAPDH, M_3_ receptors and TRPV1

Gene	Primer(5′-3′)
*GAPDH - F*	TGAAGCAGGCATCTGAGGG
*GAPDH - R*	CGAAGGTGGAAGAGTGGGAG
*M*_*3*_ *- F*	AGCGCCTTCTGTCTGCTTTTG
*M*_*3*_ *- R*	CAGCCCTCTCTGATCTCGCC
*TRPV1 - F*	CAAGGCTCTATGATCGCAGG
*TRPV1 - R*	GAGCAATGGTGTCGTTCTGC

### Statistical analysis

Data were expressed as mean values ± standard error of the mean (SEM). Statistical analyses were processed using SPSS 19.0 software (SPSS Inc., Chicago, IL, USA). Digital images were assessed using the Image-Pro Plus. 6.0. Results were analysed using one-way ANOVA. A probability of *P* < 0.05 was considered statistically significant.

## Results

### General findings

The fasting blood glucose (FBG) level of the group II mice was significantly higher than that of the group I mice after 4 times of STZ intraperitoneal injection combined with HFD feeding (*P* < 0.01) (Table 3), indicating that the diabetic mouse model was successfully established.

**Table 3:** FBG in model and age matched control mice before and after treatment

Group	0 week	2 weeks	Group	15 weeks	16 weeks	17 weeks	18 weeks
Group I	96.5 ± 1.3	98.6 ± 2.9	Control	99.2 ± 2.7	102.6 ± 3.3	101.3 ± 2.8	108.9 ± 3.4
Group II	95.4 ± 1.4	196.9 ± 17.7^**^	Model	214.7 ± 36.0^**^	191.9 ± 24.3^**^	214.7 ± 36.0^**^	245.5 ± 28.0^**^
			Positive	181.6 ± 23.4	197.3 ± 24.5	181.6 ± 23.4	226.4 ± 29.5
			HRL	186.1 ± 22.2	211.3 ± 23.1	186.1 ± 22.2	204.1 ± 21.5
			LRL	202.5 ± 31.8	170.1 ± 34.6	202.5 ± 31.8	196.0 ± 34.3

0 week: The time before the first STZ injection; 2 weeks: the time after injection with STZ for 4 times; 15-18 weeks: the time when diabetic mice received the treatment of RL or Methycobal. Group I: control mice (*n* = 10); Group II: type 2 diabetes mellitus model mice (*n* = 60). After the type 2 diabetes mellitus mouse model was established, Group II mice were randomly divided into model, positive, HRL and LRL groups (n = 8). Model vs. control group ^**^P < 0.01

**Fig. 1:**
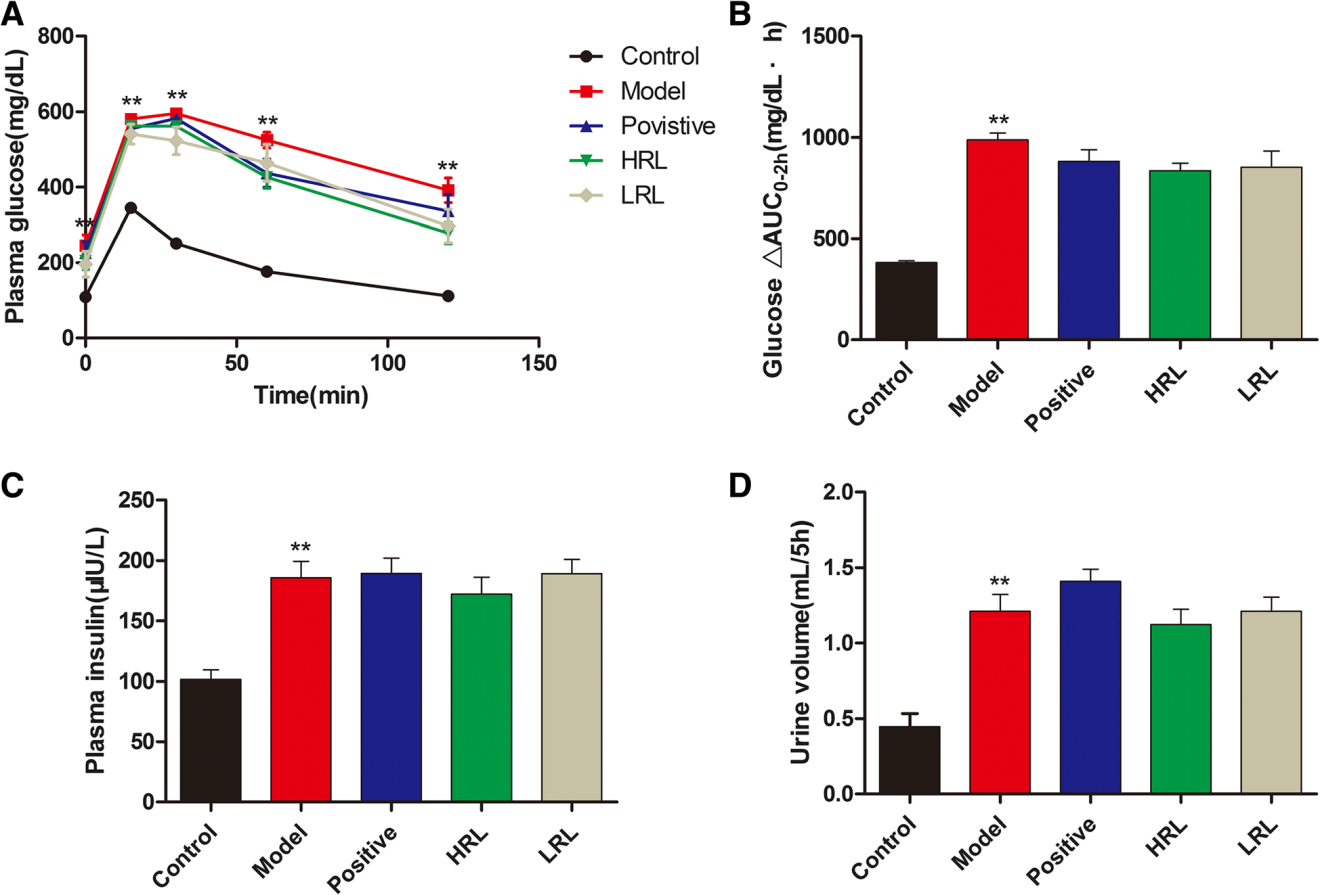
Effects of RL on mice glucose metabolism and urine volume after treatment for 4 weeks (*n* = 8). **a** OGTT (Mice were fasted overnight and fed with D-glucose at 2 g/kg body weight, plasma glucose was measured at 0, 15, 30, 60, and 120 min after glucose challenge), (**b**) Area under the curve (AUC_0-2h_) of the blood glucose (Bg) time course from 0 to 120 min (Formula: AUC0-2 h = (Bg0 + Bg15) × 0.5 ÷ 4 + (Bg15 + Bg30) × 0.5 ÷ 4 + (Bg30 + Bg60) × 0.5 ÷ 2 + (Bg60 + Bg120) × 0.5), (**c**) Plasma insulin levels and (**d**) 5 h urine volume in five groups mice. Model vs. control group ^**^P < 0.01

The later decompensated phase of DBD was induced by 12 weeks of persistent hyperglycemia, and then, the mice were received 4 weeks of treatment. However, no significant differences in FBG, OGTT, plasma insulin, glucose AUC_0–2h_ and 5 h urine volume were observed among the HRL, LRL, and model groups (Table 3, Fig. 1a, b, c, and d).

### Urodynamic test in vivo

**Fig. 2:**
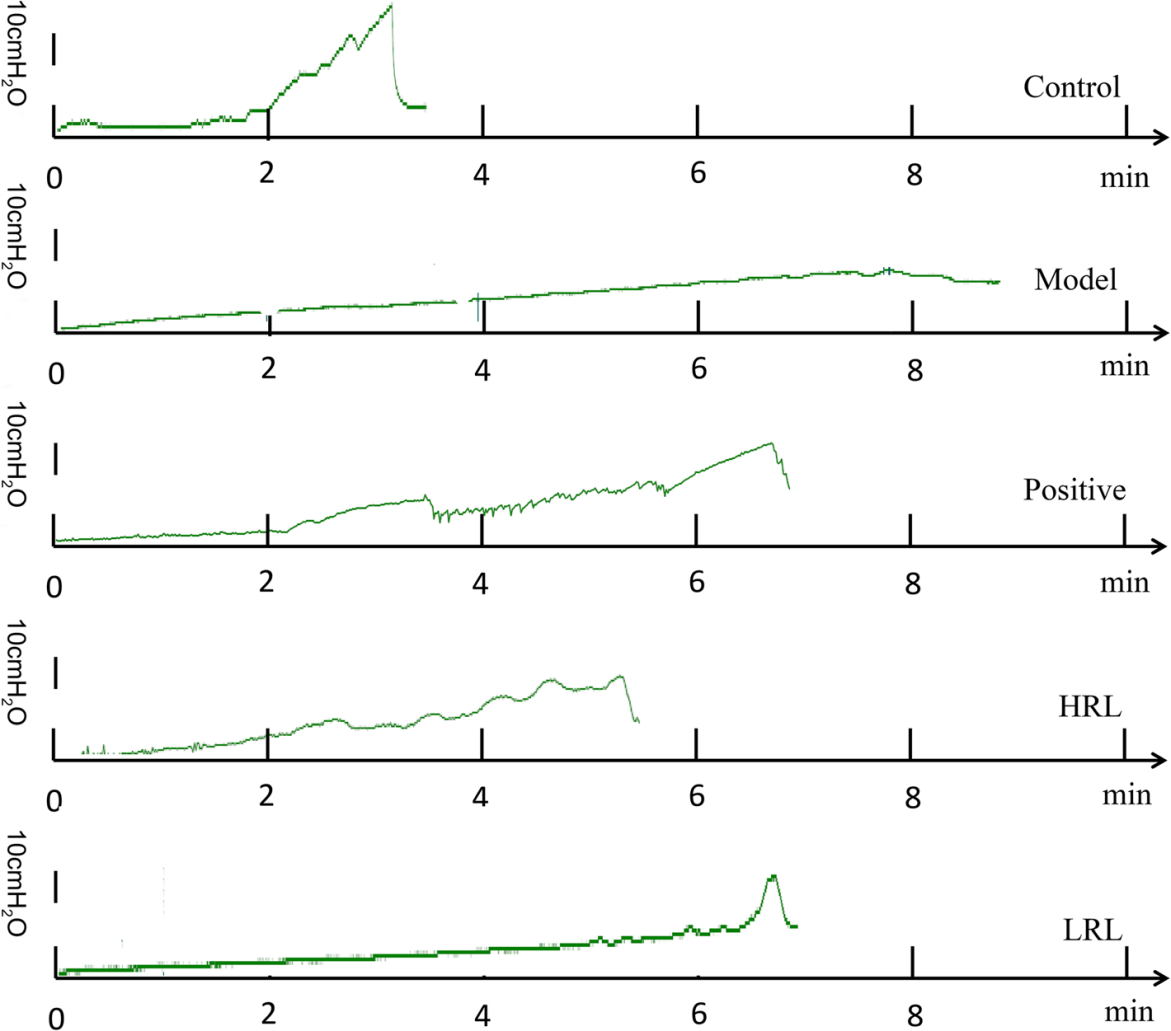
Representative original recordings of urodynamic test from five groups mice

**Fig. 3:**
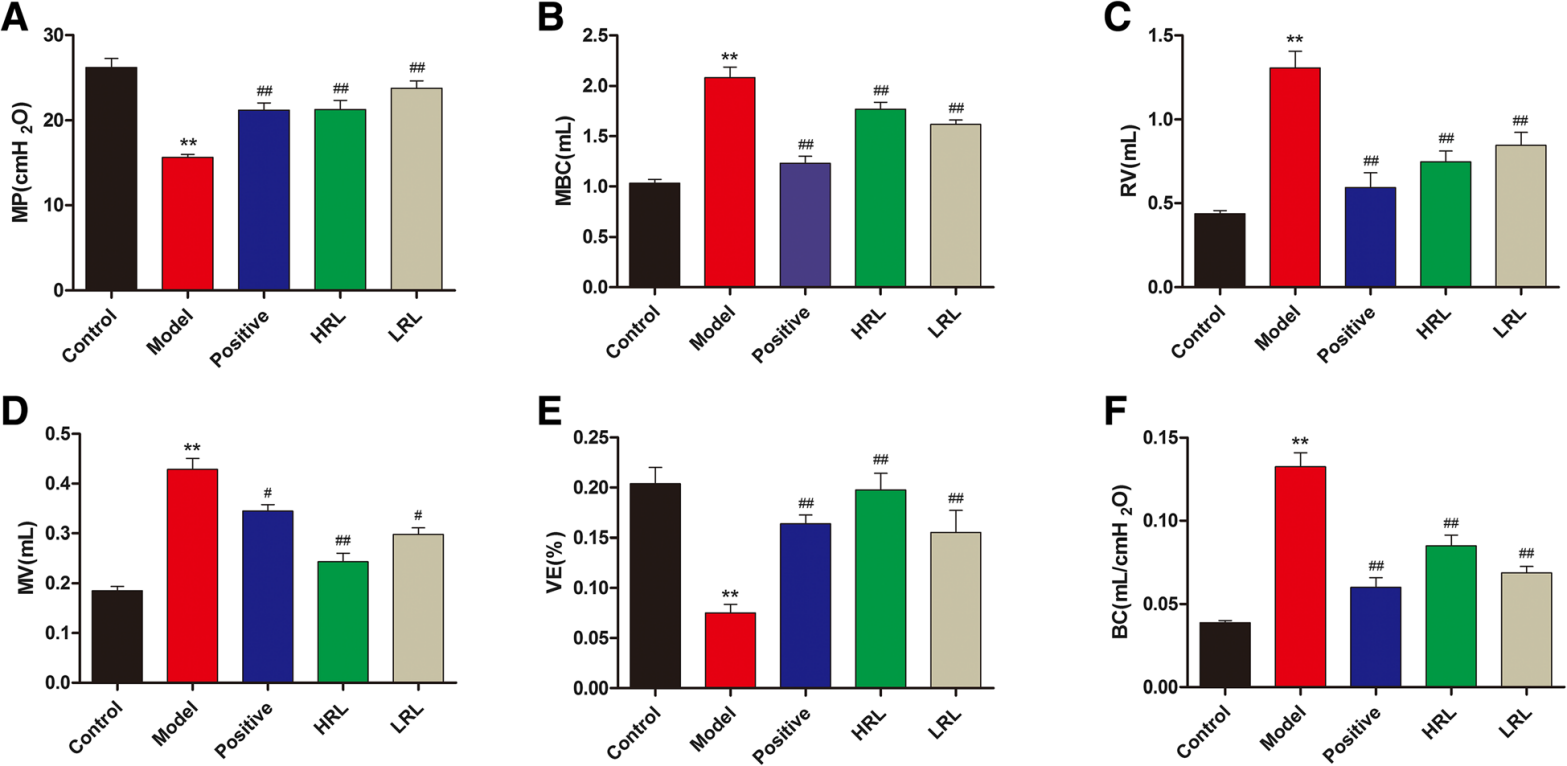
Representative cystometric recording from all groups of mice (*n* = 8). Urodynamic detection measured after 4 weeks of treatment included (**a**) maximum voiding pressure (MP), (**b**) maximum bladder capacity (MBC), (**c**) residual volume (RV), (**d**) micturition volume (MV), (**e**) voided efficiency (VE) and (**f**) bladder compliance (BC). MBC was measured as the amount of saline infused into the bladder until urination. RV was drained manually and measured using a 1 ml syringe. VE was calculated as (MBC – RV)/MBC × 100%. BC was calculated as (MBC/MP) × 100%. Model vs. control group, ^*^P < 0.05 and ^**^P < 0.01; Treatment vs. model group, ^#^*P* < 0.05 and ^##^*P* < 0.01

Urodynamic test results showed that the MV, MP and VE of the model group mice significantly decreased (*P* < 0.01), whereas the MBC, RV and BC markedly increased (*P* < 0.01), compared with those of the control group mice, showing the typical phenomenon of later decompensated phase in the bladder. After RL treatment, the VE remarkably elevated, and the MBC, RV and BC were remarkably decreased (*P* < 0.01). Furthermore, HRL remarkably increased the MV (*P* < 0.05). In addition, no significant differences in the MP among the HRL, LRL, and model groups (Fig. 2, Fig. 3a, b, c, d, e and f).

### Histological test

**Fig. 4:**
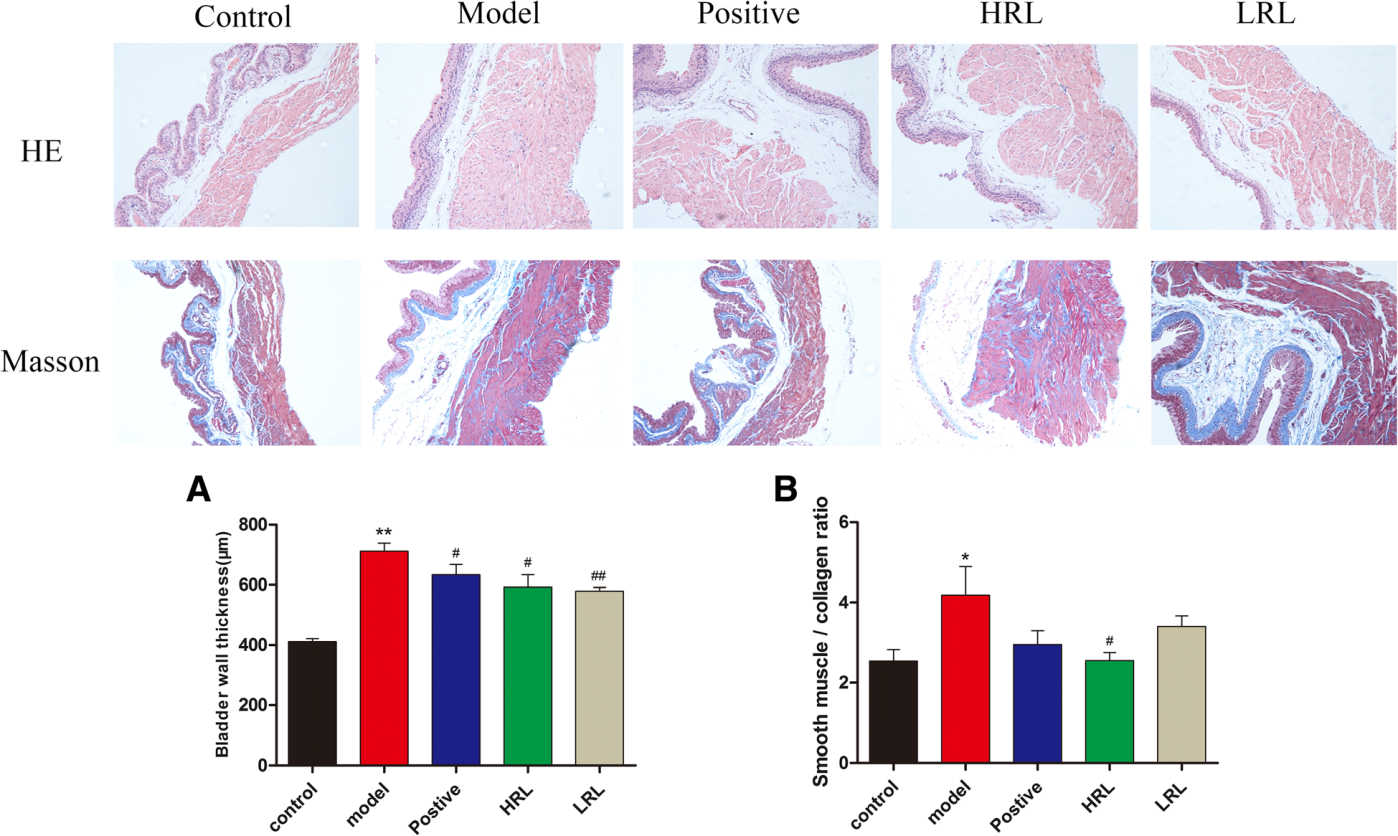
Digitalization images (100×) from H&E staining and Masson’s trichrome staining. (**a**) Bladder wall thickness was measured on H&E images (*n* = 8). (**b**) The ratio of smooth muscle to collagen within bladder smooth muscle layer wall was determined using the images stained with Masson’s trichrome (*n* = 7). Model vs. control group ^*^P < 0.05 and ^**^P < 0.01; Treatment vs. model group ^#^*P* < 0.05 and ^##^*P* < 0.01

Bladder wall thickness was measured on H&E images. The ratio of smooth muscle to collagen in the smooth muscle layer was determined using images stained with Masson’s trichrome. Morphometric analysis of bladder tissues revealed that the bladder wall thickness and the ratio (smooth muscle/collagen) significantly increased (*P* < 0.01 and *P* < 0.05, respectively) in model group mice, as compared with the control mice. HRL decreased the bladder wall thickness and the ratio of smooth muscle/collagen (*P* < 0.05) (Fig. 4a and b). The bladder wall thickness also remarkably decreased after LRL treatment for 4 weeks (*P* < 0.01) (Fig. 4a and b).

### Effect of stimulus on the bladder smooth muscle contraction

Each bladder strip was allowed to balance for 45 min before response. α, β-methylene ATP (100 μM), CAP (10 μM), KCl (120 mM) and carbachol (10^− 8^ M to 10^− 5^ M) were used to induce bladder contractions (Representative original recordings of the contraction were shown in Additional file 2).

**Fig. 5:**
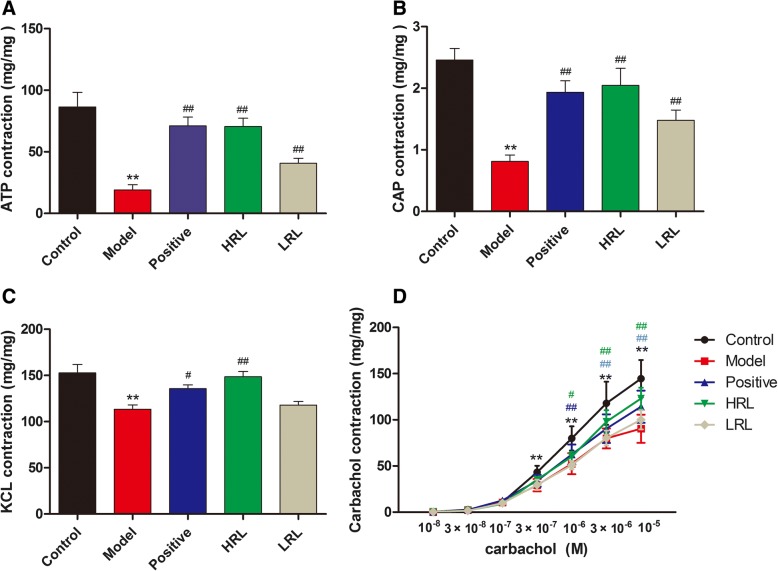
In vitro contractile responses of bladder detrusor from all groups of mice to four stimuli (n = 8), including (**a**) α,β-methylene ATP (100 μM), (**b**) CAP (10 μM), (**c**) KCl (120 mM) and (**d**) carbachol (10^− 8^ M to 10^− 5^ M.). Model vs. control group, ^**^P < 0.01; Treatment vs. model group, ^#^*P* < 0.05 and ^##^*P* < 0.01

Maximum contractions of the bladder strip response to α, β-methylene ATP, CAP, KCl and carbachol were significantly lower in the model group (*P* < 0.01) than in control group. By contrast, treatment with HRL and LRL significantly improved the bladder strip responses to α, β-methylene ATP, CAP, KCL and carbachol (*P* < 0.01 or *P* < 0.05) (Fig. 5a, b, c and d).

### Gene expression levels of M_3_ receptors and TRPV1

**Fig. 6:**
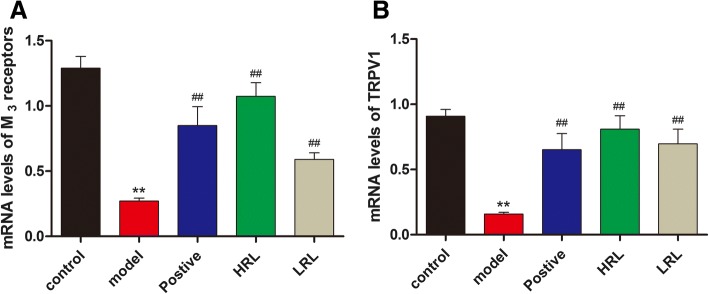
Effect of HRL or LRL treatment on the role of (**a**) M_3_ receptors, and (**b**) TRPV1 in the bladder of model mice (*n* = 8). Model vs. control group, ^**^P < 0.01; Treatment vs. model group. ^#^*P* < 0.05 and ^##^*P* < 0.01

Real-time PCR results showed that, as compared with control group, the gene expression levels of M_3_ receptors and TRPV1 were significantly down-regulated in the model group (*P* < 0.01). HRL or LRL treatment up-regulated the gene expression levels of M_3_ receptors and TRPV1 (*P* < 0.01), compared with the model mice (Fig. 6a, b).

## Discussion

Since the limitation of the therapeutic method in clinical, a number of high-efficient pharmacologic natural products were used to treat diabetes and its complications. Radix Linderae, a traditional Chinese medicine, was also a kind of natural product, which has great effect in treating bladder dysfunction through reducing symptoms of urinary frequency and/or urgency, urinary incontinence and other bladder dysfunction [24].

Diabetic bladder dysfunction (DBD) was one of the main complications of diabetes affecting 80% of patients with diabetes [3]. However, the molecular mechanisms of DBD remained elusive and treatment options were limited. In specific, the later decompensated phase of DBD was associated with multiple cystopathic complications, including impaired bladder sensation, decreased detrusor contractility, increased capacity and RV [25], which would cause intense pain to the patient.

Streptozotocin (STZ) - induced diabetic mice or rats usually produced abnormal blood glucose, and the major urodynamic alterations in those mouse model exhibited similar symptoms as humans clinically [26, 27]. However, the alterations were time-dependent [28] and varied from the species or even strain of animal. The results from our general and urodynamic studies confirmed that the C57BL/6J model mice showed higher FBG, OCTT, plasma insulin, glucose AUC_0–2h_, urine volume and most of the bladder complications (increased MBC, BC, RV and decreased VE) produced in humans in the later decompensated phase of DBD [29]. RL treatment for 4 weeks did not significantly improve the blood glucose levels in the diabetic mice but apparently improved the voiding capacity of model mice, suggesting that the therapeutic effect of RL was directly target on the bladder rather than improving blood glucose levels. In addition, in this study, we have obtained the reliable data efficiently from the single cystometrogram (CMG) to reflect the functional change of diabetes bladder and prove the therapeutic effects of RL on the later phase of DBD. At the same time, we also realized that continuous CMG can provide more comprehensive and detailed data.

Furthermore, some studies reported that the structural remodelling also occurred in STZ-induced diabetes model mice [30, 31]. Poladia et al. quantified the size and composition of bladder detrusor, and found that the bladder wall in model mice increased by a 2-fold, which mainly contributed to the growth of the bladder smooth muscle layer. Thus, smooth muscle hypertrophy and/or hyperplasia maybe closely related to the changes in urodynamic alterations. Imbalance in the muscle-to-collagen ratio would lead to subsequent bladder dysfunction in diabetic mice, and the collagen content gradually decreased as the DBD progresses [26]. In our study, the histological analysis revealed the significant alterations in diabetic bladder detrusor wall structure, which was consistent with previous studies [26, 31]. The increased bladder detrusor wall thickness in model mice was in line with the increased bladder capacity, and the enhanced of muscle-to-collagen ratio in the smooth muscle layer resulted in increased MBC, BC, RV and decreased VE. After 4 weeks treatment, RL not only decreased the of bladder wall thickness, but also reduced the ratio of smooth muscle to collagen within the bladder smooth muscle layer wall. These results, along with the urodynamic test data showing the hyporesponsiveness in the model mice, revealed an impaired bladder voiding function of model mice. Thus, RL can clearly improve the bladder detrusor structure and voiding function.

The function of bladder detrusor contractility was mainly regulated by cholinergic pathways, and carbachol was commonly chosen as an exogenous neurotransmitter to activate M_3_ receptors, causing contraction [32, 33]. Furthermore, purinergic and TRPV1 pathways also played important roles in bladder contraction, especially in DBD [34, 35]. In vitro functional tests of isolated bladder detrusor muscle obtained from diabetic mice revealed time-dependent cystometric alterations. In the early phase, the responses of detrusor strips to α, β-methylene ATP, CAP, carbachol and KCl were increased, whereas the responses were decreased in the later phase. This theory of DBD has been described in STZ-induced diabetic mice, rats and gene knock-out diabetic mice [3, 18, 28]. In the present study, the contractions of the model mice to exogenous muscarinic, TRPV1 and purine receptor agonists (carbachol, CAP and α, β-methylene ATP, respectively) significantly reduced, which matched the results of urodynamic test in vivo and histological test. By contrast, the bladder strips responses markedly increased in the HRL or LRL groups, indicating that RL may be worked on those related receptors. Moreover, the KCl-induced tension response various from different groups, indicating the alteration of bladder smooth muscle, which could be another reason contributed to the tension change of carbachol, CAP and α, β-methylene ATP.

In the bladders of DBD mice, we found lower gene expressions of M_3_ receptors, which was in line with other studies [36]. This result suggested that the voiding capacity and bladder hyporesponsiveness in the later phase of DBD decreased through the down-regulation of M_3_ receptors gene expression. RL extracts up-regulated the number of M_3_ receptors in the bladder which could be one of the mechanisms in DBD treatment. TRPV1 activation could enhance bladder contraction [37]. The gene expression of TRPV1 in bladder-innervating dorsal root ganglia neurons was observed in STZ-induced diabetes rat model at 4 weeks after STZ injection, and bladder detrusor strips contractions response to CAP markedly decreased [35]. Our results were in line with this study in bladder detrusor strips contractions response to CAP, which was significantly decreased. However, the gene expression of TRPV1 was significantly decreased in the DBD mice bladder tissue. This discrepancy can be attributed to the different strain and disease phase. Furthermore, the TRPV1 gene expression in the RL-treated mouse bladders markedly up-regulated, suggesting that this up-regulation contributed to the therapeutic effect.

## Conclusions

Our data were consistent with the hypothesis and offered convincing evidence, in vivo and in vitro, that RL ethanol extracts can protect the bladder function from the later phase of DBD. It not only effectively improved the bladder voiding function, bladder wall tissue structure, and detrusor contractility of model mice but also regulated gene expression of M_3_ receptors and TRPV1 in model mouse bladder. These novel findings provide insights into the possible mechanisms of RL in treatment and offered credibility for further clinical application. The mechanisms by which this modulation occurred were not clear, but worthy of further investigation.

## Additional files

Additional file 1: The quality control of Radix Linderae (RL). (DOCX 165 kb)

Additional file 2: Original recordings of the detrusor strips contraction to stimulus. (DOCX 595 kb)

## Abbreviations

ANOVA: analysed using one-way
BC: bladder compliance
CAP: capsaicin
DBD: diabetic bladder dysfunction
FBG: fasting blood glucose
HFD: high fat diet
HRL: high-dose RL
LRL: low-dose RL
MBC: maximum bladder capacity
MP: maximum voiding pressure
MV: micturition volume
OGTT: oral glucose tolerance test
RL: Radix Linderae
RV: residual volume
SEM: standard error of the mean
STZ: streptozotocin
T2DM: type 2 diabetes mellitus
TRPV1: transient receptor potential vanilloid-1
VE: voided efficiency
VSOP: voided stain on paper

## References

[1] Yuan Z, Tang Z, He C, Tang W (2015). Diabetic cystopathy: a review. J Diabetes.

[2] Daneshgari F, Liu G, Birder L, Hannamitchell AT, Chacko S (2009). Diabetic bladder dysfunction: current translational knowledge. J Urol.

[3] Wang Z, Cheng Z, Cristofaro V, Li J, Xiao X, Gomez P, Ge R, Gong E, Strle K, Sullivan MP (2012). Inhibition of TNF-alpha improves the bladder dysfunction that is associated with type 2 diabetes. Diabetes.

[4] Brown JS, Wessells H, Chancellor MB, Howards SS, Stamm WE, Stapleton AE, Steers WD, Sk VDE, Mcvary KT (2005). Urologic complications of diabetes. Diabetes Care.

[5] Liu RT, Chung MS, Lee WC, Chang SW, Huang ST, Yang KD, Chancellor MB, Chuang YC (2011). Prevalence of overactive bladder and associated risk factors in 1359 patients with type 2 diabetes. Urology.

[6] Golbidi S, Laher I (2010). Bladder dysfunction in diabetes mellitus. Front Pharmacol.

[7] Andersson KE, Arner A (2004). Urinary bladder contraction and relaxation: physiology and pathophysiology. Physiol Rev.

[8] Liu G, Daneshgari F (2005). Alterations in neurogenically mediated contractile responses of urinary bladder in rats with diabetes. Am J Physiol Renal Physiol.

[9] Branchini E, Ursino E, Corsi A, Martizzi D, Amati L, Herder JWD, Galeazzi M, Gendre B, Kaastra J, Moscardini L (2009). Studying the warm hot intergalactic medium with gamma-ray bursts. Astrophys J.

[10] Kobayashi S, Yamagami T, Kadowaki SI, Ishida T, Schep C, Borg H, Shoel Kobayashi, et al.: Disc-shaped recording medium, Disc driving device and disc producing method. 2005.

[11] Ding RB, Tian K, Huang LL, He CW, Jiang Y, Wang YT, Wan JB (2012). Herbal medicines for the prevention of alcoholic liver disease: a review. J Ethnopharmacol.

[12] Xu YF, Liang ZJ, Kuang ZJ, Chen JJ, Wu J, Lu XE, Jiang WW, Fan PL, Tang LY, Li YT (2017). Effect of Suo Quan Wan on the bladder function of aging rats based on the beta-adrenoceptor. Exp Ther Med.

[13] Lai H, Yan Q, Cao H, Chen P, Xu Y, Jiang W, Wu Q, Huang P, Tan B (2016). Effect of SQW on the bladder function of mice lacking TRPV1. BMC Complement Altern Med.

[14] Lai H, Tan B, Liang Z, Yan Q, Lian Q, Wu Q, Huang P, Cao H (2015). Effect of the Chinese traditional prescription Suo Quan Wan on TRPV1 expression in the bladder of rats with bladder outlet obstruction. BMC Complement Altern Med.

[15] Cheng XL, Ma SC, Wei F, Wang GL, Xiao XY, Lin RC (2007). A new sesquiterpene isolated from Lindera aggregata (SIMS) KOSTERM. Chem Pharm Bull.

[16] Wu Y, Zheng Y, Liu X, Han Z, Ren Y, Gan L, Zhou C, Luan L (2010). Separation and quantitative determination of sesquiterpene lactones in Lindera aggregata (Wu-Yao) by ultra-performance LC-MS/MS. J Sep Sci.

[17] Wang C, Dai YJ, Chou G, Wang C, Wang Z (2007). Treatment with total alkaloids from Radix Linderae reduces inflammation and joint destruction in type II collagen-induced model for rheumatoid arthritis. J Ethnopharmacol.

[18] Daneshgari F, Liu G, Imrey PB (2006). Time dependent changes in diabetic cystopathy in rats include compensated and decompensated bladder function. J Urol.

[19] de Oliveira MG, Nascimento DM, Alexandre EC, Bonilla-Becerra SM, Zapparoli A, Monica FZ, Antunes E. Menthol ameliorates voiding dysfunction in types I and II diabetic mouse model. Neurourol Urodyn. 2018. 10.1002/nau.23785 30088676

[20] Mahzari A, Zeng XY, Zhou X, Li S, Xu J, Tan W, Vlahos R, Robinson S, Ye JM. Repurposing matrine for the treatment of hepatosteatosis and associated disorders in glucose homeostasis in mice. Acta Pharmacol Sin. 2018. 10.1038/s41401-018-0016-8 PMC6289344 29980742

[21] Wang JW, Chen XY, Pei-Yang HU, Tan MM, Tang XG, Huang MC, Lou ZH (2016). Effects of Linderae radix extracts on a rat model of alcoholic liver injury. Exp Ther Med.

[22] Wang Z, Cheng Z, Cristofaro V, Li J, Xiao X, Gomez P, Ge R, Gong E, Strle K, Sullivan MP (2012). Inhibition of TNF-α improves the bladder dysfunction that is associated with type 2 diabetes. Diabetes.

[23] Alexandre EC, Calmasini FB, de Oliveira MG, Silva FH, Da SC, André DM, Leonardo FC, Delbin MA, Antunes E (2016). Chronic treatment with resveratrol improves overactive bladder in obese mice via antioxidant activity. Eur J Pharmacol.

[24] Schoendorfer N, Sharp N, Seipel T, Schauss AG, Ahuja KDK (2018). Urox containing concentrated extracts of Crataeva nurvala stem bark, Equisetum arvense stem and Lindera aggregata root, in the treatment of symptoms of overactive bladder and urinary incontinence: a phase 2, randomised, double-blind placebo controlled trial. BMC Complement Altern Med.

[25] Daneshgari F, Liu G, Birder L, Hanna-Mitchell AT, Chacko S (2009). Diabetic bladder dysfunction: current translational knowledge. J Urol.

[26] Gray MA, Wang CC, Sacks MS, Yoshimura N, Chancellor MB, Nagatomi J (2008). Time-dependent alterations of select genes in streptozotocin-induced diabetic rat bladder. Urology.

[27] Wang T, Li X, Zhou B, Li H, Zeng J, Gao W. Anti-diabetic activity in type 2 diabetic mice and α-glucosidase inhibitory, antioxidant and anti-inflammatory potential of chemically profiled pear peel and pulp extracts (Pyrus spp.). J Funct Foods. 2015;13:276–88.

[28] Daneshgari F, Huang X, Liu G, Bena J, Saffore L, Powell CT (2006). Temporal differences in bladder dysfunction caused by diabetes, diuresis, and treated diabetes in mice. Am J Physiol.

[29] Brown JS, Nyberg LM, Kusek JW, Burgio KL, Diokno AC, Foldspang A, Fultz NH, Herzog AR, Hunskaar S, Milsom I (2003). Proceedings of the National Institute of Diabetes and Digestive and Kidney Diseases international symposium on epidemiologic issues in urinary incontinence in women. Am J Obstet Gynecol.

[30] Pitre DA, Ma T, Wallace LJ, Bauer JA (2002). Time-dependent urinary bladder remodeling in the streptozotocin-induced diabetic rat model. Acta Diabetol.

[31] Poladia DP, Bauer JA (2005). Functional, structural, and neuronal alterations in urinary bladder during diabetes: investigations of a mouse model. Pharmacology.

[32] Cheng JT, Yu BC, Tong YC (2007). Changes of M3-muscarinic receptor protein and mRNA expressions in the bladder urothelium and muscle layer of streptozotocin-induced diabetic rats. Neurosci Lett.

[33] Leiria LO, Mónica FZ, Carvalho FD, Claudino MA, Franco-Penteado CF, Schenka A, Grant AD, De NG, Antunes E (2011). Functional, morphological and molecular characterization of bladder dysfunction in streptozotocin-induced diabetic mice: evidence of a role for L-type voltage-operated Ca^2+^ channels. Br J Pharmacol.

[34] Avelino A, Cruz F (2006). TRPV1 (vanilloid receptor) in the urinary tract: expression, function and clinical applications. Naunyn Schmiedeberg’s Arch Pharmacol.

[35] Sharopov BR, Gulak KL, Philyppov IB, Sotkis AV, Shuba YM. TRPV1 alterations in urinary bladder dysfunction in a rat model of STZ-induced diabetes. Life Sci. 2017:193. 10.1016/j.lfs.2017.10.042 29100756

[36] Pak KJ, Ostrom RS, Matsui M, Ehlert FJ (2010). Impaired M3 and enhanced M2 muscarinic receptor contractile function in a streptozotocin model of mouse diabetic urinary bladder. Naunyn Schmiedeberg’s Arch Pharmacol.

[37] Dinis P, Charrua A, Avelino A, Yaqoob M, Bevan S, Nagy I, Cruz F (2004). Anandamide-evoked activation of vanilloid receptor 1 contributes to the development of bladder hyperreflexia and nociceptive transmission to spinal dorsal horn neurons in cystitis. J Neurosci.

