# Supplementary material for: Effects of Radix Linderae extracts on a mouse model of diabetic bladder dysfunction in later decompensated phase
Source: BMC Complement Altern Med. 2019 Feb 4;19:41. doi: 10.1186/s12906-019-2448-1 (PMC6360732; doi:10.1186/s12906-019-2448-1)
Supplement: Supplementary file 1 — The quality control of Radix Linderae (RL). (DOCX 165 kb) [file 12906_2019_2448_MOESM1_ESM.docx]

**The quality control of Radix Linderae (RL)**

According to the Chinese Pharmacopeia, RL should contain more than 0.03% of linderane and more than 0.40% of norisoboldine. Our determination result showed that the RL we used met the requirements.

1. *HPLC determination of linderane*

Authentication of linderane is provided in Table 1. Linderane was calculated according to the dry product, and the content was 0.63%.

**Table S1** HPLC chromatography of linderane

| Filling agent | Mobile phase | Spectrum |
| --- | --- | --- |
| Octadecylsilane Chemically bonded silica | Acetonitrile–water  (56:44) | 235 nm |


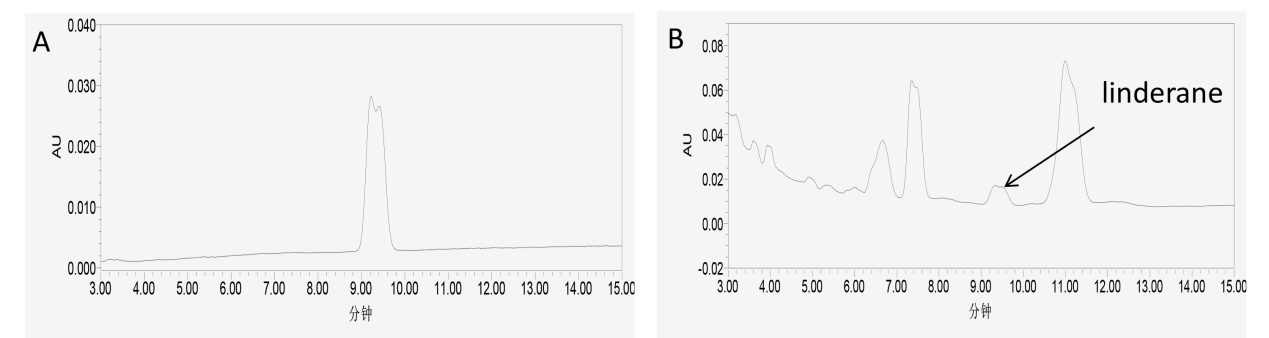
 **Figure S1**

**Figure S1** HPLC chromatography of RL. (A) Standard substance of linderane. (B) Chromatography of RL.

1. *HPLC determination of norisoboldine*

Authentication of norisoboldine is provided in **Table S2**. Norisoboldine was calculated according to the dry product, and the content was 0.58%.

**Table S2** HPLC chromatography of norisoboldine.

| Filling agent | Mobile phase A | Mobile phase B | Spectrum |
| --- | --- | --- | --- |
| Octadecylsilane Chemically bonded silica | Acetonitrile | 0.5% methanoic acid and  0.1% triethylamine solution | 280 nm |


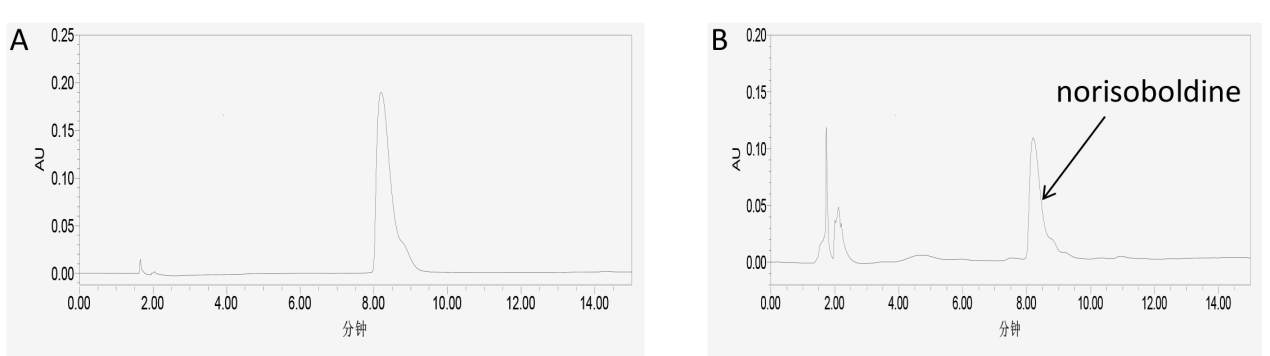


**Figure S2**

**Figure S2** HPLC chromatography of RL. (A) Standard substance of norisoboldine. (B) Chromatography of RL.
