# Supplementary material for: Effects of Radix Linderae extracts on a mouse model of diabetic bladder dysfunction in later decompensated phase
Source: BMC Complement Altern Med. 2019 Feb 4;19:41. doi: 10.1186/s12906-019-2448-1 (PMC6360732; doi:10.1186/s12906-019-2448-1)
Supplement: Supplementary file 2 — Original recordings of the detrusor strips contraction to stimulus. (DOCX 595 kb) [file 12906_2019_2448_MOESM2_ESM.docx]

**Original recordings of the detrusor strips contraction to stimulus**
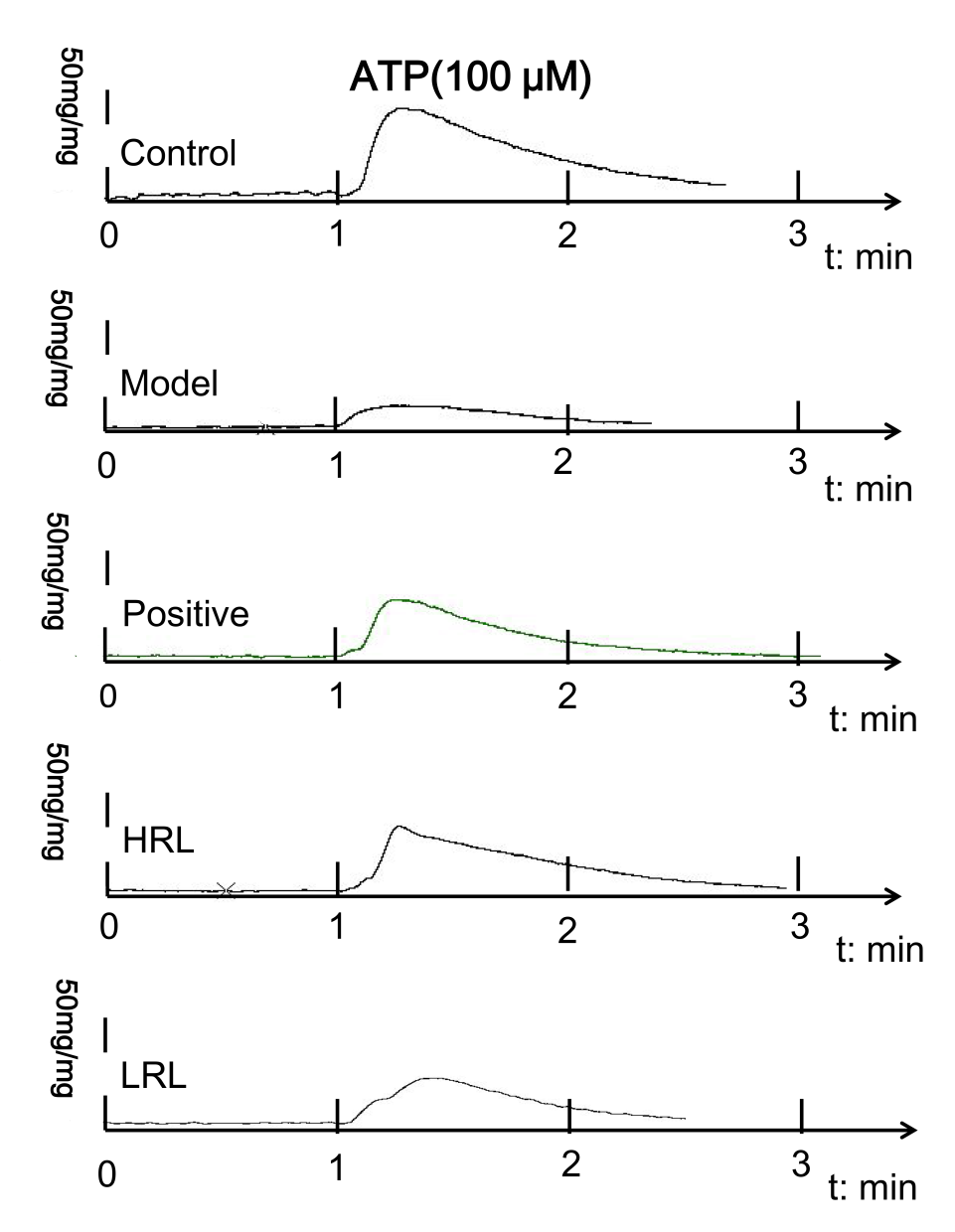


**(A)**


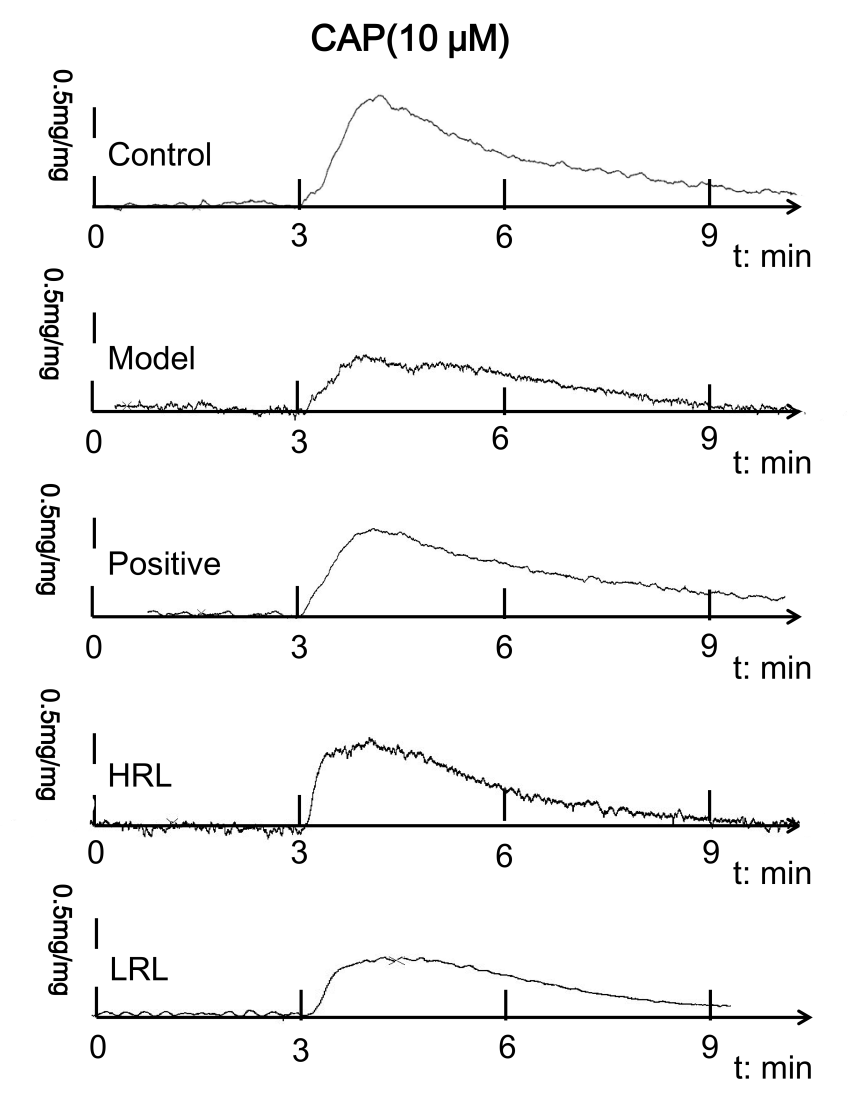


**(B)**


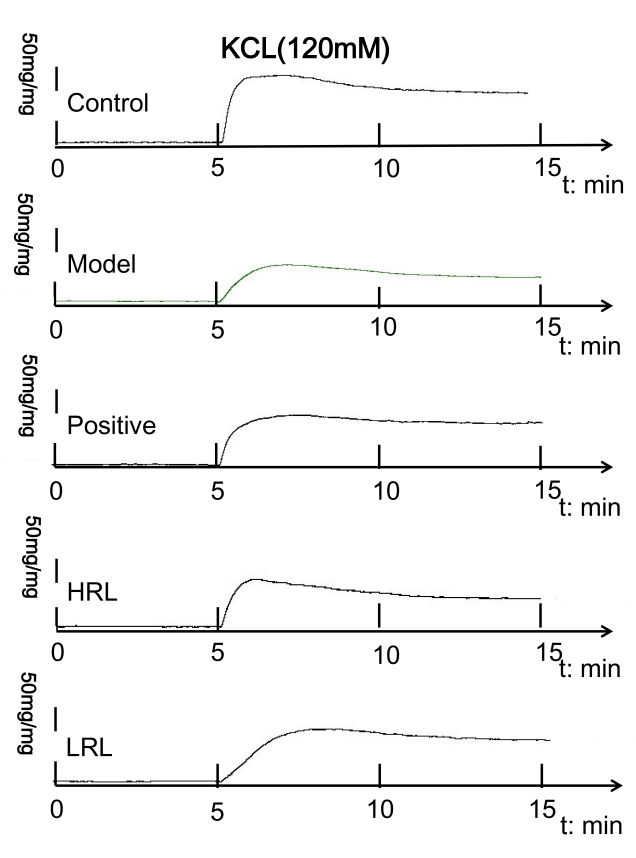


**(C)**


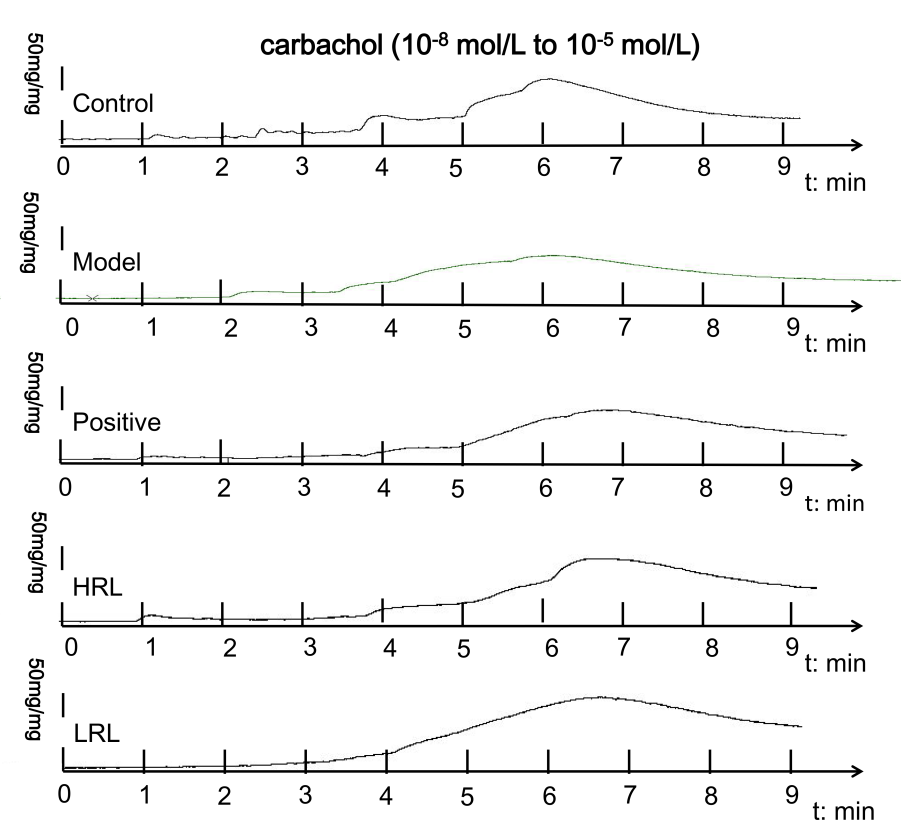


**(D)**

**Figure S3** *In vitro* contractile responses of bladder detrusor from all groups of mice to four stimuli (n = 8), including (A) α,β-methylene ATP (100 μM), (B) CAP (10 µM), (C) KCl (120 mM) and (D) carbachol (10^-8^ M to 10^-5^ M.).
